# Metabolic tumour area: a novel prognostic indicator based on ^18^F-FDG PET/CT in patients with diffuse large B-cell lymphoma in the R-CHOP era

**DOI:** 10.1186/s12885-024-12668-x

**Published:** 2024-07-25

**Authors:** Silu Cui, Wenchong Xin, Fei Wang, Xiaoliang Shao, Xiaonan Shao, Rong Niu, Feifei Zhang, Yunmei Shi, Bao Liu, Weiying Gu, Yuetao Wang

**Affiliations:** 1 Department of Nuclear Medicine, The Third Affiliated Hospital of Soochow University, Changzhou, Jiangsu China; 2 Institute of Clinical Translation of Nuclear Medicine and Molecular Imaging, Soochow University, Changzhou, Jiangsu China; 3 Yangzhou University, Yangzhou, Jiangsu China; 4 Department of Nuclear Medicine, Linyi People’s Hospital, Linyi, Shandong China; 5 Department of Hematology, The Third Affiliated Hospital of Soochow University, Changzhou, Jiangsu China

**Keywords:** ^18^F-FDG PET/CT, Diffuse large B-cell lymphoma, Total metabolic tumour volume, Prognosis, Metabolic tumour area

## Abstract

**Background:**

The metabolic tumour area (MTA) was found to be a promising predictor of prostate cancer. However, the role of MTA based on ^18^F-FDG PET/CT in diffuse large B-cell lymphoma (DLBCL) prognosis remains unclear. This study aimed to elucidate the prognostic significance of MTA and evaluate its incremental value to the National Comprehensive Cancer Network International Prognostic Index (NCCN-IPI) for DLBCL patients treated with first-line R-CHOP regimens.

**Methods:**

A total of 280 consecutive patients with newly diagnosed DLBCL and baseline ^18^F-FDG PET/CT data were retrospectively evaluated. Lesions were delineated via a semiautomated segmentation method based on a 41% SUVmax threshold to estimate semiquantitative metabolic parameters such as total metabolic tumour volume (TMTV) and MTA. Receiver operating characteristic (ROC) curve analysis was used to determine the optimal cut-off values. Progression-free survival (PFS) and overall survival (OS) were the endpoints that were used to evaluate the prognosis. PFS and OS were estimated via Kaplan‒Meier curves and compared via the log-rank test.

**Results:**

Univariate analysis revealed that patients with high MTA, high TMTV and NCCN-IPI ≥ 4 were associated with inferior PFS and OS (*P* < 0.0001 for all). Multivariate analysis indicated that MTA remained an independent predictor of PFS and OS [hazard ratio (HR), 2.506; 95% confidence interval (CI), 1.337–4.696; *P* = 0.004; and HR, 1.823; 95% CI, 1.005–3.310; *P* = 0.048], whereas TMTV was not. Further analysis using the NCCN-IPI model as a covariate revealed that MTA and NCCN-IPI were still independent predictors of PFS (HR, 2.617; 95% CI, 1.494–4.586; *P* = 0.001; and HR, 2.633; 95% CI, 1.650–4.203; *P* < 0.0001) and OS (HR, 2.021; 95% CI, 1.201–3.401; *P* = 0.008; and HR, 3.869; 95% CI, 1.959–7.640; *P* < 0.0001; respectively). Furthermore, MTA was used to separate patients with high NCCN-IPI risk scores into two groups with significantly different outcomes.

**Conclusions:**

Pre-treatment MTA based on ^18^F-FDG PET/CT and NCCN-IPI were independent predictor of PFS and OS in DLBCL patients treated with R-CHOP. MTA has additional predictive value for the prognosis of patients with DLBCL, especially in high-risk patients with NCCN-IPI ≥ 4. In addition, the combination of MTA and NCCN-IPI may be helpful in further improving risk stratification and guiding individualised treatment options.

**Trial registration:**

This research was retrospectively registered with the Ethics Committee of the Third Affiliated Hospital of Soochow University, and the registration number was approval No. 155 (approved date: 31 May 2022).

**Supplementary Information:**

The online version contains supplementary material available at 10.1186/s12885-024-12668-x.

## Introduction

Diffuse large B-cell lymphoma (DLBCL) is the most common subtype of adult non-Hodgkin lymphoma (NHL), accounting for approximately 25% of adult cases of NHL [1]. Despite the overall improvement in the prognosis of DLBCL, especially with the advent of rituximab, cyclophosphamide, doxorubicin, vincristine, and prednisone (R-CHOP) chemoimmunotherapy, up to 30–50% of patients are refractory to R-CHOP or relapse after achieving complete response (CR) [2, 3]. Thus, early identification of patients with relapsed/refractory DLBCL is of great importance, the significance is essential because low-risk patients can be cured with the current standard R-CHOP regimen; in contrast, high-risk patients may benefit from intensive treatment or new therapies [4]. The international prognostic index (IPI) is a basic tool used for the risk stratification of DLBCL patients and has been widely used by clinicians. However, the predictive value of the IPI has been questioned because R-CHOP or R-CHOP-like regimens have become routine treatments for DLBCL [5, 6]. In addition, research has demonstrated that the National Comprehensive Cancer Network-IPI (NCCN-IPI), a new prognostic scoring system, outperforms the IPI in predicting outcomes and improving the risk stratification of DLBCL [7]. Nonetheless, current prognostic scoring systems, based solely on clinically relevant parameters, are insufficient to identify high-risk populations with poor outcomes exactly [7–9]. As a result, efforts are still needed to develop more meaningful prognostic markers, such as imaging indicators, to identify high-risk patients the NCCN-IPI cannot distinguish.

Guidelines [10–13] recommended that 18-fluorodeoxyglucose positron emission computed tomography (^18^F-FDG PET/CT) be routinely used for the diagnosis, staging, restaging and posttreatment response assessment of lymphoma. More accurate staging means patients can choose superior therapy, leading to longer survival. The semiquantitative metabolic parameters obtained by ^18^F-FDG PET/CT, typically the SUVmax, total metabolic tumour volume (TMTV) and total lesion glycolysis (TLG), have attracted increasing interest from researchers and clinicians. Most studies [14–16] have demonstrated the prognostic significance of metabolic parameters in DLBCL. However, relevant studies [17, 18] claim that the TMTV has limited predictive value for DLBCL. Therefore, published studies on the prognostic value of these parameters are still controversial.

Previous articles [19–21] have shown that the tumour size and length of the largest lesion measured via ^18^F-FDG PET/CT can rapidly, simply, and objectively predict the prognosis of DLBCL. Ryuichi Mizuno et al. [22] investigated the prognostic significance of a novel indicator, the metabolic tumour area (MTA), in prostate cancer. Although multivariate analysis revealed that MTA was not an independent predictor of PSA relapse, considering its accessibility and strong prognostic value, similar to MTV in univariate analysis, we speculate that MTA may have equal prognostic value in DLBCL patients. As a result, we aimed to investigate the prognostic value of MTA in DLBCL, which may be more meaningful than tumour length in assessing the extent of bulky disease. To our knowledge, it has never been evaluated in patients with DLBCL.

The purpose of this study was to (1) explore and develop novel and readily available semiquantitative indicators that reflect the metabolic burden of tumour from pre-treatment ^18^F-FDG PET/CT and (2) evaluate the prognostic value of MTA in whole DLBCL patients and subgroups with a high risk of NCCN-IPI score ≥ 4.

## Materials and methods

### Patients

**Fig. 1:**
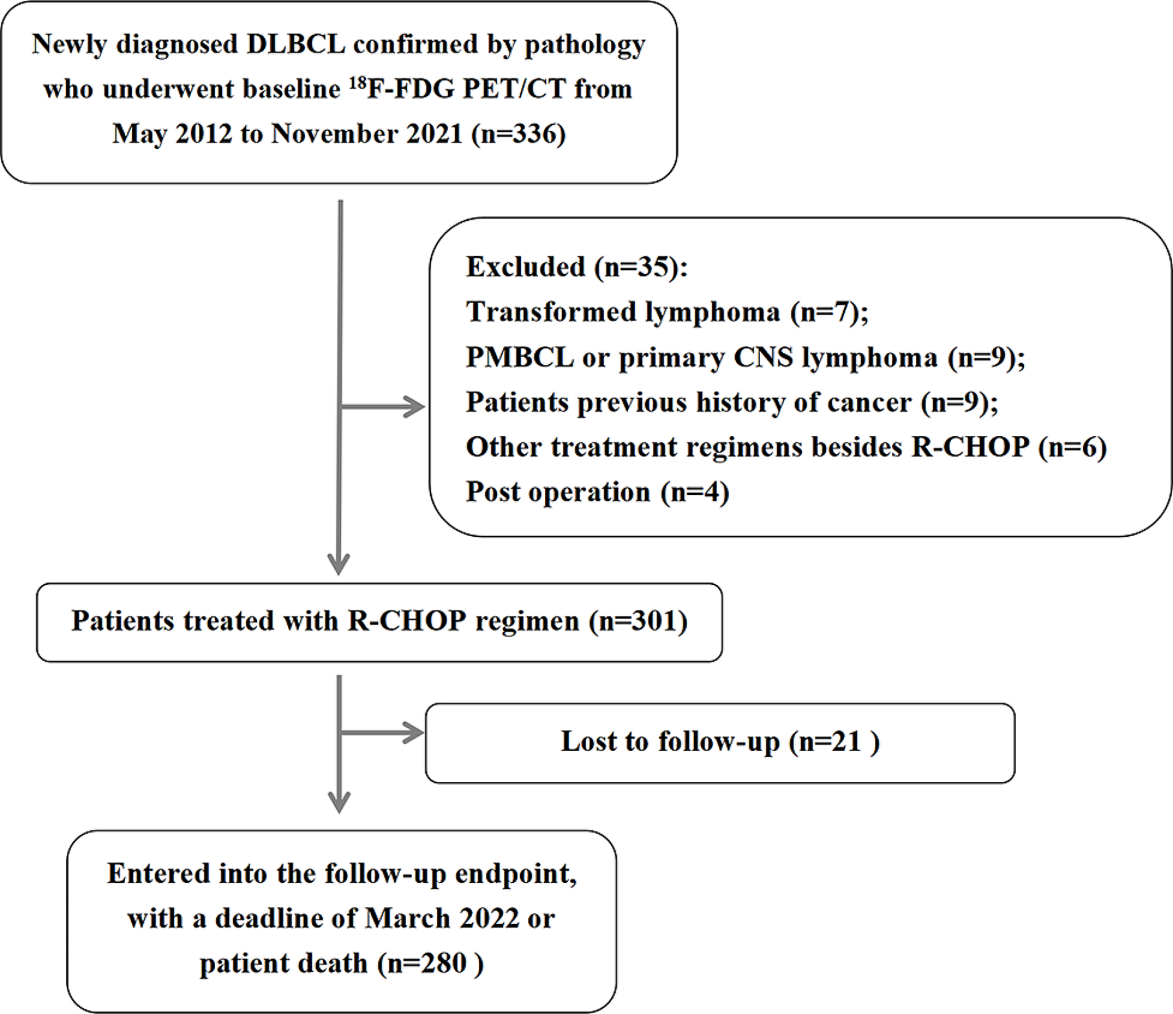
Flow chart of patient selection (PMBCL: primary mediastinal B-cell lymphoma; CNS: central nervous system)

Patients with newly diagnosed DLBCL who underwent baseline ^18^F-FDG PET/CT scans at the Third Affiliated Hospital of Soochow University from May 2012 to November 2021 were retrospectively analysed in our study. The inclusion criteria were as follows: (1) de novo-diagnosed DLBCL patients who met the WHO’s pathological classification criteria, (2) had baseline ^18^F-FDG PET/CT scans before treatment, and (3) were receiving first-line treatment with the R-CHOP regimen. The exclusion criteria were as follows: (1) transformed lymphoma, (2) primary mediastinal B-cell lymphoma (PMBCL), (3) primary central nervous system (CNS) lymphoma, (4) lack of serum lactate dehydrogenase (LDH) levels, (5) treatment before ^18^F-FDG PET/CT, and (6) other treatment regimens in addition to R-CHOP (Fig. 1).

Baseline clinical characteristics were collected from medical records, including Lugano stage, age, serum LDH level, Eastern Cooperative Oncology Group performance status (ECOG PS), Ann arbor stage and extranodal involvement (EN) sites. In addition to the Lugano stage, the other five baseline clinical characteristics were used to calculate the NCCN-IPI score as follows: age 41–60 years (1 point), 60–75 years (2 points), > 75 years (3 points); LDH > 1 time the upper limit of normal (ULN) (1 point), > 3 times the ULN (2 points); Ann arbor stage III/IV and ECOG PS ≥ 2 (1 point); and EN in major organs (1 point). The total score for the NCCN-IPI ranges from 0 to 8 points. Four distinct risk groups were then formed based on the scores: low risk (scores 0–1), low–intermediate risk (scores 2–3), high–intermediate risk (scores 4–5) and high risk (scores 6–8). Patients were further grouped into low-risk patients with an NCCN-IPI < 4 and high-risk patients with an NCCN-IPI ≥ 4, which was proposed by Zhou et al. [7] in 2014 and written into the NCCN guidelines in 2016.

### ^**18**^**F-FDG PET/CT acquisition**

All patients underwent whole-body ^18^F-FDG PET/CT image acquisition on a German Siemens Biograph CT (64) PET/CT machine. Data such as height, weight, and plasma glucose level were recorded, and patient plasma glucose levels did not exceed 11 mmol/L. Six hours of fasting preceded the examination, then ^18^F-FDG was administered intravenously, depending on the patient’s body weight, at 3.70–5.55 MBq/kg. The radiopharmaceutical was provided by Nanjing Jiangyuan Andico Positron Research and Development Company (radiochemical purity > 95%). Whole-body PET/CT scans were started after the participants rested in a quiet and comfortable environment for 60 min. Data were acquired at a rate of 2 min/bed position, and the scan range was from the top of the skull to the upper thigh. Each patient received approximately 6–7 scans. The CT data for attenuation correction and anatomical localisation were acquired after the PET/CT scan. The specific parameters for CT scanning were as follows: the tube voltage was 120 kV, the tube current was 35 mA, the pitch was 0.8, the rotation time was 0.5 s, and the layer thickness was 5 mm. After image acquisition, the ordered subset expectation maximisation (OSEM) method was used for PET image reconstruction (two iterations, 21 subsets). The acquired images from the PET and CT scans were sent for image registration and fusion via the postprocessing workstation TrueD system (Siemens) to obtain the reconstructed PET transverse, sagittal, coronal tomographic and PET/CT fusion images.

### ^**18**^**F-FDG PET/CT image analysis**

**Fig. 2:**
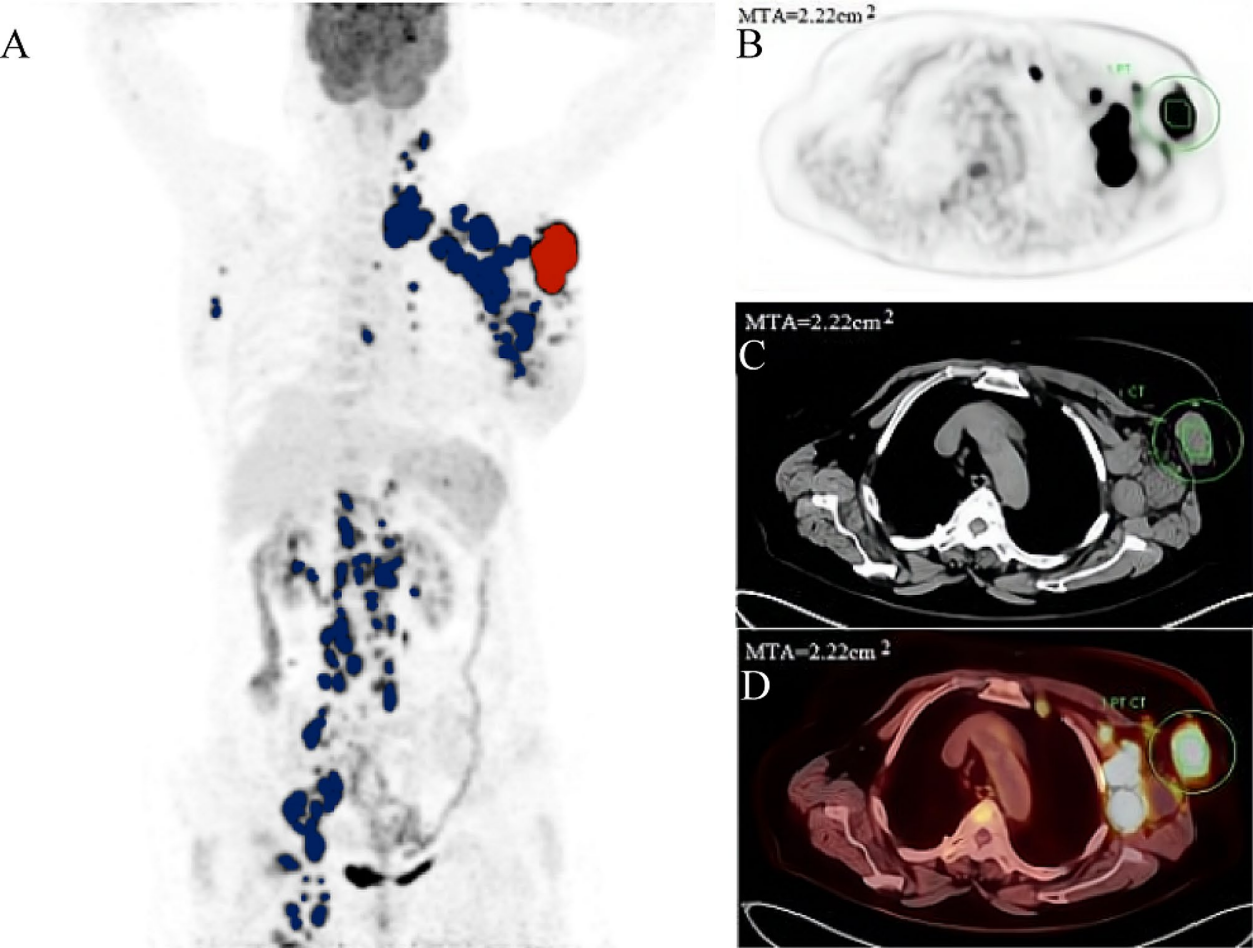
Delineation and calculation of the MTA based on ^18^F-FDG PET/CT images. A: Maximum intensity projection (MIP) is used to identify the largest lesion. All involved lesions are shown in blue, and the largest lesions used to obtain MTA are represented in red. B: PET tomographic reconstruction of cross-sectional images. C: CT tomographic reconstruction of cross-sectional images. D: The maximum cross-sectional area of the largest lesion on the ^18^F-FDG PET/CT tomographic fusion images to obtain the MTA, which can be automatically displayed in the TrueD system and delineated by a solid yellow line, with an MTA of 2.22 cm^2^

Two experienced nuclear medicine physicians blinded to patient outcomes analysed the PET/CT images. The results of abnormal lesions were determined by consensus. PET image data were exported from the Siemens workstation in DICOM format and uploaded into LIFEx software (version 5.10, http://www.lifexsoft.org) [23]. Regions of interest (ROIs) were delineated using a 41% SUVmax threshold to compute the TMTV, MTA, SUVmax and SUVmean, as the European Association of Nuclear Medicine (EANM) guidelines recommended [24]. The highest SUV and the mean SUV for all lesions were the final SUVmax and SUVmean, respectively. The TMTV was defined as the sum of the metabolic volumes of all measurement lesions. MTA was defined as the maximum cross-sectional area of the largest lesion on the ^18^F-FDG PET/CT cross-section. The nuclear medicine physicians clicked on the projection of each hypermetabolic lesion in the graphical user interface, and the system automatically generated the contour around the target lesion within the boundary and defined the metabolic area with voxels within the contour boundary presenting a 41% SUVmax threshold. The maximum value was recorded as the MTA, which can be automatically displayed in the TrueD system (Fig. 2).

For each PET dataset, physiologic uptake (e.g., bladder, kidney, brain and myocardium) and false-positive lesions (e.g., inflammation and infection) were removed and missed tumour regions were added. Additionally, the nontumour regions adjacent to the tumour were manually deleted slice-by-slice by an eraser tool if necessary. The bone marrow (BM) and liver were only measured if there was focal uptake, whereas the spleen was considered involved if there was focal uptake or diffuse uptake > 150% of the liver background [25]. A visual assessment was performed to confirm that only pathological lesions were included.

### Follow-Up and endpoints

After completing the standard R-CHOP regimen, patients were followed up by telephone and retrospective review of medical records until March 2022 or when the patient died. All patients’ survival status was confirmed by checking death records, by calling immediate family members, or by the patient himself. The recently revised criteria for response assessment in patients with malignant lymphoma were used to determine the timing of disease progression or relapse [10, 11]. Disease relapse was defined as clinical or imaging evidence of relapse and then confirmed during follow-up. Progression-free survival (PFS) and overall survival (OS) were selected as endpoints for estimating the prognosis of patients with DLBCL. PFS was calculated from the documented date of diagnosis until the date of disease progression (new lesion or enlargement of the previous existing lesion), relapse or death from the disease. OS was estimated from the date of diagnosis to the date of death from any cause or the last follow-up.

### Statistical analysis

This study’s statistical analyses were performed via IBM SPSS (version 19.0; SPSS Inc., Chicago, Illinois, USA). Continuous variables are expressed as the mean ± standard deviation (SD, normal distribution) or median (Q1–Q3, skewed distribution). Categorical variables are expressed as frequencies or rates (%). Differences in clinical data and PET/CT metabolic parameters between different groups (binary variables) were tested via the χ^2^ test (for categorical variables), T-test (for normally distributed data), or Mann‒Whitney U test (skewed distribution). Pearson’s correlation coefficient was used to evaluate the correlation between the above indicators. For each PET-derived feature, we conducted receiver operating characteristic (ROC) analysis to determine the optimal cut-off values for predicting the occurrence of an event (PFS or OS) by maximising the Youden index. Then we calculated the sensitivity and specificity of the cut-off value. The distributions of the 3-year PFS rate and OS rate were estimated via Kaplan‒Meier curves and the survival curves of the different groups were compared via the log-rank test. Univariate and multivariate analyses (MVAs) were performed via the Cox proportional hazards model. Cox regression was used to estimate the hazard ratio (HR) and confidence interval (CI). The intraclass correlation coefficient (ICC) was used to analyse the consistency of the measurement data between two observers. A two-tailed *P* value < 0.05 was considered statistically significant.

## Results

### Patient characteristics

A total of 280 patients with newly diagnosed DLBCL whose clinical characteristics and PET/CT quantification metrics are summarised in Table 1 were included in the study. The sample included 135 (48.2%) females and 145 (51.8%) males. The median age was 69 years (13–87 years). Among these patients, progression/relapse occurred in 98 (35%) patients at a median of 20.5 months (ranging from 0 to 88 months), and death occurred in 63 (22.5%) patients at a median of 23 months (ranging from 0 to 61 months). The 3-year PFS and OS rates for the whole group were 63.1% and 75.9%, respectively.

**Table 1:** Characteristics of the 280 enrolled patients

Characteristic	value
**Gender**,** n (%)**	
Male	145 (51.8)
Female	135 (48.2)
**Age (years)**,** n (%)**	
≤ 40	21 (7.5)
41–≤60	68 (24.3)
61–≤75	134 (47.9)
> 75	57 (20.4)
*EN,** n (%)**	
0–1	166 (59.3)
> 1	114 (40.7)
**Ann Arbor Stage**,** n (%)**	
Stage I/II	115 (41.1)
Stage III/IV	165 (58.9)
**Lugano Stage**,** n (%)**	
Stage I/II	105 (37.5)
Stage III/IV	175 (62.5)
**LDH**,** n (%)**	
Normal (≤ 250)	153 (54.6)
High (> 250)	127 (45.4)
**ECOG PS**,** n (%)**	
0–1	195 (69.6)
> 1	85 (30.4)
**NCCN-IPI**,** n (%)**	
Low (0/1)	36 (12.9)
Low–intermediate (2/3)	93 (33.2)
High–intermediate (4–5)	102 (36.4)
High (6–8)	49 (17.5)
**MTA (cm** ^**2**^ **)**	
Mean (SD)	4.49 (5.70)
Median (range)	2.37 (0.15–29.58)
**TMTV (cm** ^**3**^ **)**	
Mean (SD)	199.40 (303.21)
Median (range)	65.85 (0.58–2057.09)
**SUVmax (g/ml)**	
Mean (SD)	32.37 (21.90)
Median (range)	30.59 (2.71–225.11)
**SUVmean (g/ml)**	
Mean (SD)	11.15 (8.15)
Median (range)	8.65 (0.86–53.85)

Abbreviations: EN, extranodal involvement; LDH, lactate dehydrogenase; ECOG, Eastern Cooperative Oncology Group; NCCN-IPI, National Comprehensive Cancer Network-International Prognostic Index; MTA, metabolic tumour area; TMTV, total metabolic tumour volume; SD, standard deviation

*Involvement of major organs (bone marrow, central nervous system, liver, gastrointestinal tract, or lung)

### Correlations between MTA and other metabolic parameters, NCCN-IPI and components of the NCCN-IPI

Pearson’s correlation analysis revealed a moderate correlation between MTA and TMTV, LDH and NCCN-IPI (ρ = 0.557, 0.514 and 0.442, respectively, *P* < 0.0001). Based on the above observations, we further calculate a variance inflation factor (VIF) to assess collinearity among the above variables. The results indicated no multicollinearity between MTA, TMTV, LDH, and NCCN-IPI (VIF = 1.669, 1.555, 1.897 and 1.656, respectively). There were only weak or very weak correlations between MTA, SUVmax, and SUVmean and the other components of the NCCN-IPI (age, EN, Ann arbor stage, and ECOG PS), as shown in Supplementary Table 1. The metabolic parameters measured by ^18^F-FDG PET/CT were highly concordant between the two observers (ICC: 0.998, *P* < 0.001).

### Metabolic parameters and ROC analysis

The median SUVmax, SUVmean, TMTV and MTA were 30.59 g/ml, 8.65 g/ml, 65.85 cm^3^and 2.37 cm^2^ (Q1–Q3, 0.95–5.02 cm^2^), respectively (Table 1). ROC analysis revealed that the optimal cut-off values of MTA were 1.36 cm^2^ for PFS and 5.73 cm^2^ for OS (areas under the ROC curve (AUCs) of 0.649 and 0.683, respectively; *P* < 0.0001), as described in Table 2 and Supplementary Fig. 1. The sensitivity/specificity values for predicting PFS and OS were 84.7%/43.4% and 42.2%/83.3%, respectively. The optimal cut-off values of the TMTV were 215.78 cm^3^ for PFS and 65.85 cm^3^ for OS (AUCs 0.658 and 0.683, respectively; *P* < 0.0001). The sensitivity/specificity values were 44.9%/80.2% and 75%/57.4% for PFS and OS, respectively. However, the AUCs of the SUVmax and SUVmean were 0.487 and 0.498 for PFS and 0.481 and 0.502 for OS, respectively (*P* > 0.05 for all).

**Table 2:** ROC analysis of metabolic parameters

Parameter	PFS						OS				
	AUC (95% CI)	Cut-off	Se	Sp	*P* value		AUC (95% CI)	Cut-off	Se	Sp	*P* value
TMTV	0.658 (0.591–0.725)	215.78	44.9	80.2	< 0.0001		0.683 (0.612–0.753)	65.85	75	57.4	< 0.0001
SUVmean	0.498 (0.429–0.567)	6.86	67.3	42	0.903		0.502 (0.425–0.579)	3.82	92.2	16.7	0.963
SUVmax	0.487 (0.419–0.555)	16.87	87.8	25.3	0.723		0.481 (0.407–0.554)	11.66	96.9	16.2	0.636
MTA	0.649 (0.583–0.715)	1.36	84.7	43.4	< 0.0001		0.683 (0.611–0.756)	5.73	42.2	83.3	< 0.0001

Se, sensitivity; Sp, specificity. The values in bold are statistically significant (*P* < 0.05)

### Outcomes according to the MTA and NCCN-IPI

**Fig. 3:**
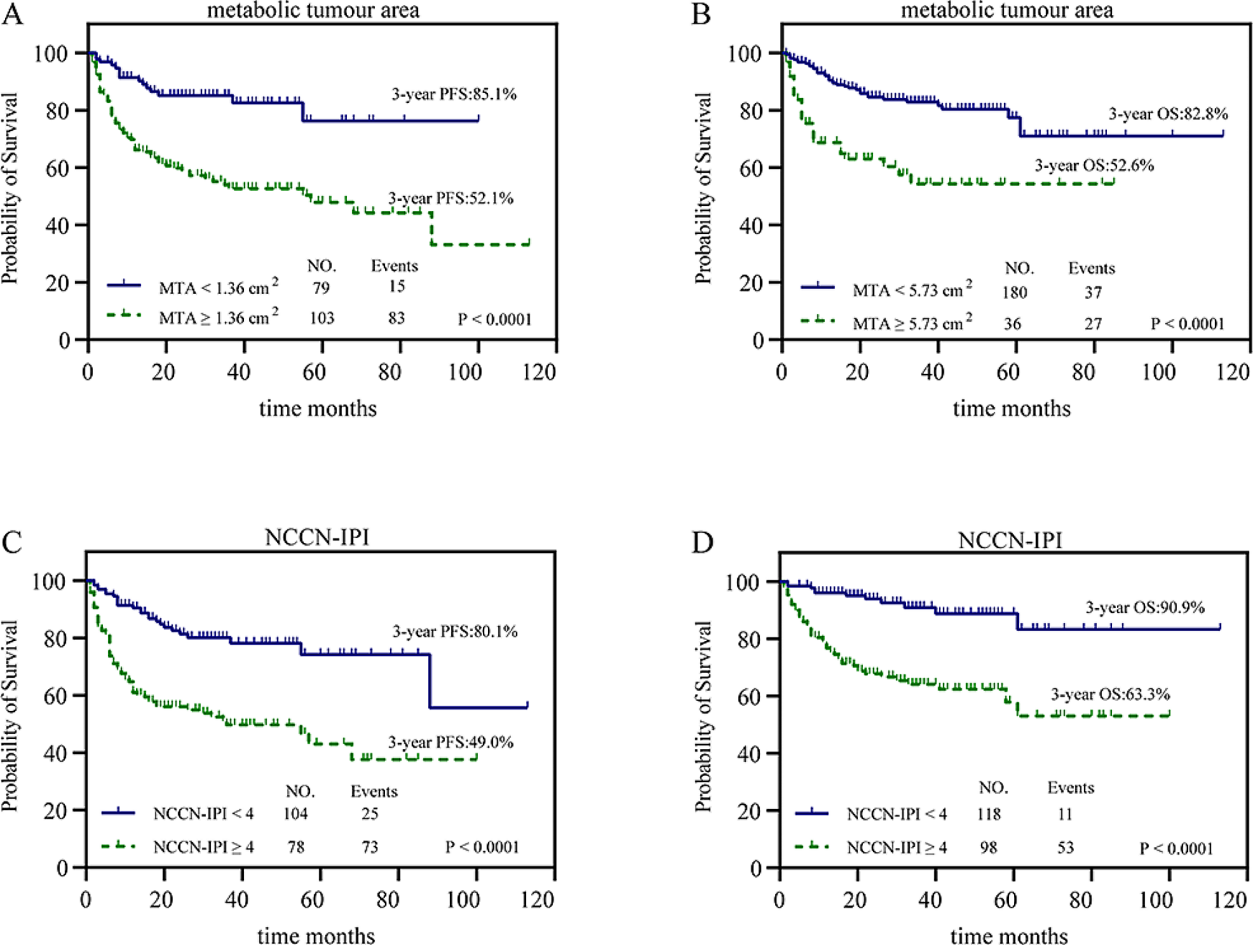
Kaplan‒Meier plots for PFS and OS in all patients in relation to MTA (< 1.36 cm^2^ vs. ≥1.36 cm^2^ and < 5.73 cm^2^ vs. ≥5.73 cm^2^, respectively) and NCCN-IPI (low-risk score 0–3 vs. high-risk score 4–8). a, b PFS (a) and OS (b) in relation to MTA. c, d PFS (c) and OS (d) in relation to the NCCN-IPI.

The median PFS and OS of patients with high MTA (≥ 1.36 cm^2^, *n* = 186 and ≥ 5.73 cm^2^, *n* = 63) were 16.5 (range, 0–113 months) and 17 months (range, 0–85 months), respectively. The median PFS and OS were 26 (range 2–100 months) and 25 months (range 1–113 months), respectively, for patients with low MTA (< 1.36 cm^2^, *n* = 94 and < 5.73 cm^2^, *n* = 217). According to the Kaplan‒Meier curves, patients with a high MTA had a 3-year PFS rate of 52.1% and an OS rate of 52.6%, whereas those with a low MTA had a 3-year PFS rate of 85.1% and an OS rate of 82.8% (both *P* < 0.0001; Fig. 3). The survival of patients with high MTA was significantly shorter than that of patients with low MTA for both PFS and OS. Patients with a low-risk NCCN-IPI of < 4 had a 3-year PFS rate of 80.1% and an OS rate of 90.9% (both *n* = 129). In contrast, those with a high-risk NCCN-IPI of ≥ 4 had a 3-year PFS rate of 49% and an OS rate of 63.3% (both *n* = 151, both *P* < 0.0001; Fig. 3).

In the Cox univariate regression analysis, the parameters statistically associated with PFS or OS were the MTA, TMTV, SUVmax, Lugano stage and components of the NCCN-IPI, whereas the SUVmean was not significant. The above indicators deemed significant in the univariate analysis were entered into a multivariate analysis, which revealed that only the MTA (HR, 2.506; 95% CI, 1.337–4.696; *P* = 0.004) and ECOG PS (HR, 1.576; 95% CI, 1.011–2.458; *P* = 0.045) remained independent predictors of PFS. In addition, the independent prognostic factors that remained meaningful for OS were age (HR, 2.110; 95% CI, 1.156–3.852; *P* = 0.015), MTA (HR, 1.823; 95% CI, 1.005–3.310; *P* = 0.048) and ECOG PS (HR, 2.107; 95% CI, 1.220–3.640; *P* = 0.008), as shown in Tables 3 and 4.

**Table 3:** Cox regression analysis for PFS

	PFS						
	Univariate analysis				Multivariate analysis		
Parameter	HR	95% CI	*P* value		HR	95% CI	*P* value
TMTV	2.620	1.753–3.914	< 0.0001		1.301	0.786–2.156	0.306
SUVmean	1.388	0.910–2.119	0.128				
SUVmax	2.106	1.151–3.856	0.016		1.478	0.792–2.755	0.219
MTA	3.354	1.934–5.817	< 0.0001		2.506	1.337–4.696	**0.004**
Lugano Stage	4.172	2.402–7.247	< 0.0001		1.476	0.570–3.823	0.423
age	1.643	1.083–2.493	0.02		1.295	0.835–2.008	0.248
Ann Arbor Stage	4.096	2.421–6.930	< 0.0001		2.403	0.946–6.105	0.065
EN	2.242	1.500–3.353	< 0.0001		1.015	0.634–1.627	0.95
LDH	2.070	1.379–3.108	< 0.0001		0.703	0.406–1.217	0.208
ECOG PS	2.360	1.582–3.523	< 0.0001		1.576	1.011–2.458	**0.045**

The values in bold are statistically significant (*P* < 0.05)

**Table 4:** Cox regression analysis for OS

	OS						
	Univariate analysis				Multivariate analysis		
Parameter	HR	95% CI	*P* value		HR	95% CI	*P* value
TMTV	3.581	2.032–6.311	< 0.0001		1.413	0.684–2.921	0.351
SUVmean	2.205	0.884–5.497	0.09				
SUVmax	5.443	1.331–22.261	0.018		2.870	0.655–12.570	0.162
MTA	3.192	1.941–5.248	< 0.0001		1.823	1.005–3.310	**0.048**
Lugano Stage	4.873	2.322–10.227	< 0.0001		1.582	0.489–5.112	0.444
age	2.682	1.520–4.732	0.001		2.110	1.156–3.852	**0.015**
Ann Arbor Stage	4.842	2.389–9.811	< 0.0001		2.270	0.736–6.998	0.154
EN	2.012	1.267–3.196	0.003		0.754	0.426–1.333	0.331
LDH	2.996	1.765–5.085	< 0.0001		1.006	0.495–2.043	0.987
ECOG PS	3.999	2.431–6.579	< 0.0001		2.107	1.220–3.640	**0.008**

The values in bold are statistically significant (*P* < 0.05)

To investigate whether MTA has incremental prognostic value for NCCN-IPI, further analysis using the NCCN-IPI model as a covariate revealed that MTA and NCCN-IPI were still independent predictors of PFS (HR, 2.617; 95% CI, 1.494–4.586; *P* = 0.001; and HR, 2.633; 95% CI, 1.650–4.203; *P* < 0.0001; respectively) and OS (HR, 2.021; 95% CI, 1.201–3.401; *P* = 0.008; and HR, 3.869; 95% CI, 1.959–7.640; *P* < 0.0001; respectively, Table 5).

**Table 5:** Multivariate analysis of MTA and NCCN-IPI in relation to PFS and OS

Parameter	PFS				OS		
	HR	95% CI	*P* value		HR	95% CI	*P* value
MTA	2.617	1.494–4.586	0.001		2.021	1.201–3.401	0.008
NCCN-IPI	2.633	1.650–4.203	< 0.0001		3.869	1.959–7.640	< 0.0001

### Outcomes according to MTA in the subgroups with low-risk and high-risk NCCN-IPI scores

**Fig. 4:**
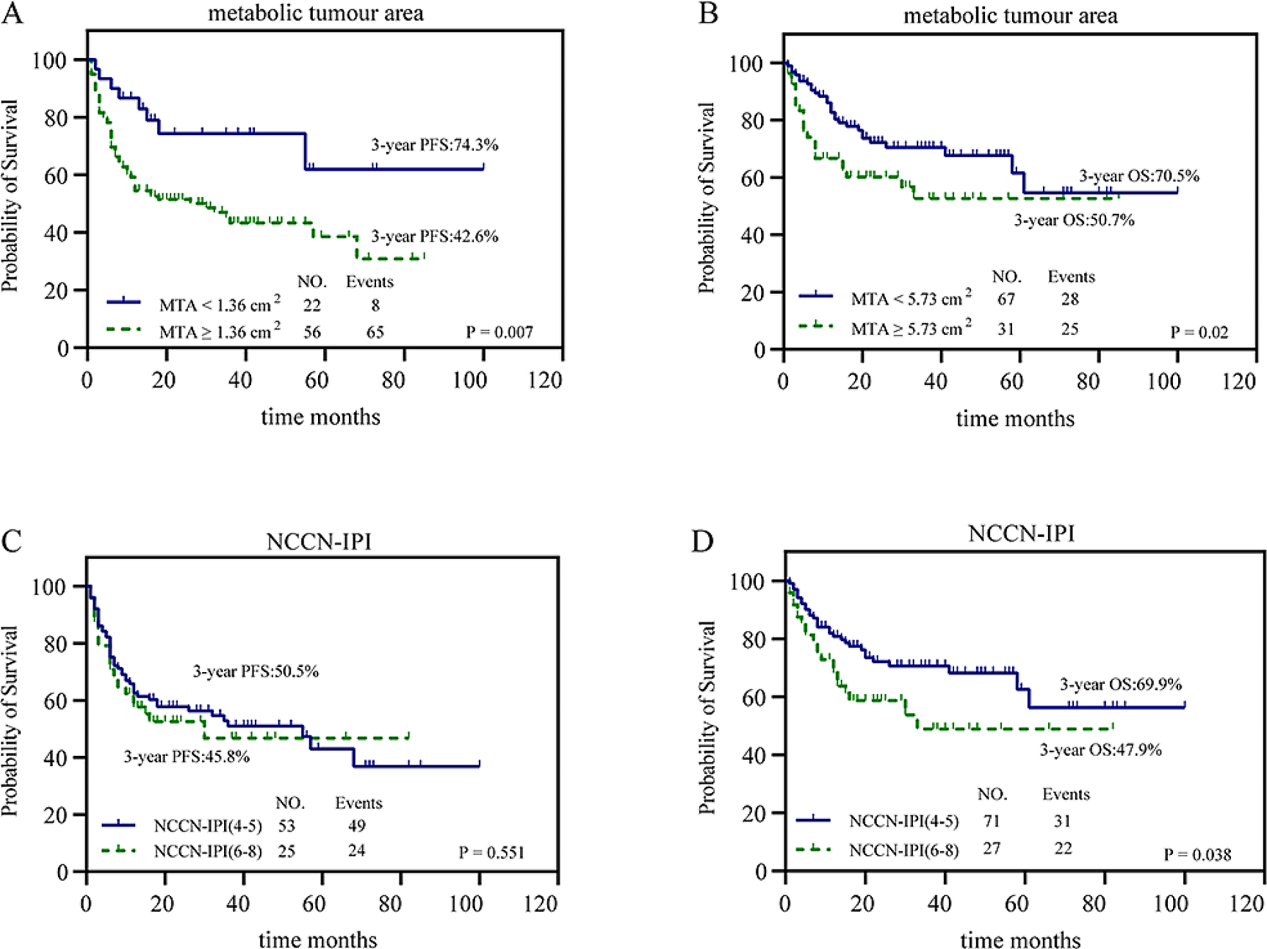
Kaplan‒Meier plots for PFS and OS in patients with a high-risk NCCN-IPI of ≥ 4 in relation to MTA (< 1.36 cm^2^ vs. ≥1.36 cm^2^ and < 5.73 cm^2^ vs. ≥5.73 cm^2^, respectively) and high-risk NCCN-IPI (high–intermediate score 4–5 vs. high score 6–8). a, b PFS (a) and OS (b) in patients with a high-risk NCCN-IPI of ≥ 4 in relation to MTA. c, d PFS (c) and OS (d) in relation to the NCCN-IPI.

**Fig. 5:**
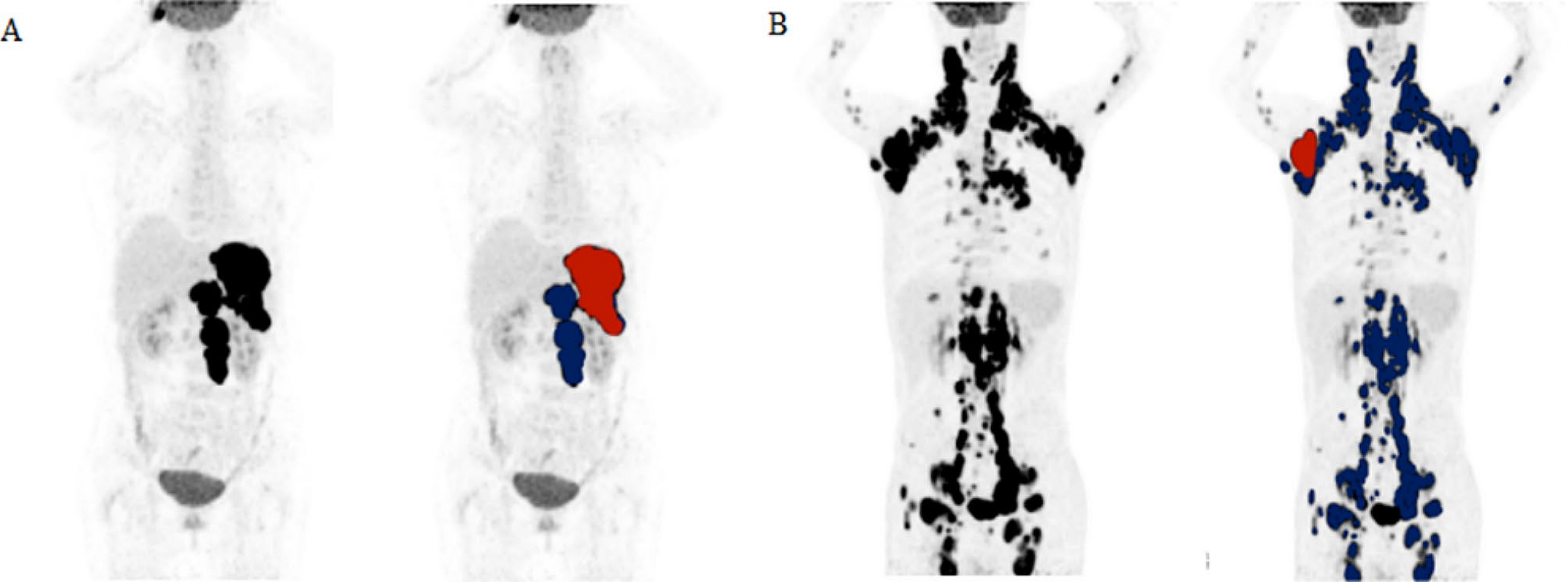
Tumour burden assessed by the TMTV and MTA based on ^18^F-FDG PET/CT and risk reclassification by the MTA in representative patients. All involved lesions are shown in blue, and the largest lesions used to obtain MTA are represented in red. (A) A patient with a high-risk NCCN-IPI (score of 6), high TMTV (273.2 cm^3)^ and high MTA (11.45 cm^2^) showed progression after two months and died three months after diagnosis. (B) A patient with a high-risk NCCN-IPI (score of 5), high TMTV (1201.55 cm^3^) and low MTA (3.64 cm^2^) showed no progression and was still alive at the end of a follow-up period of 34 months

Patients were classified into a low-risk group with an NCCN-IPI < 4 and a high-risk group with an NCCN-IPI ≥ 4. There was no significant difference in PFS between patients with an NCCN-IPI score of 4–5 and those with an NCCN-IPI score of 6–8 (*P* = 0.551), whereas the difference in OS was statistically significant (χ^2^ = 4.317, *P* = 0.038). To some extent, NCCN-IPI could separate patients with a high-risk NCCN-IPI from patients with a better outcome (*n* = 102, 50.5% 3-year PFS rate and 69.9% 3-year OS rate) and a worse outcome (*n* = 49, 45.8% 3-year PFS rate and 47.9% 3-year OS rate). On the other hand, in patients with a high-risk NCCN-IPI of ≥ 4, there were significant differences in PFS (χ^2^ = 7.265, *P* = 0.007) and OS (χ^2^ = 5.387, *P* = 0.02) between patients with low and high MTA. MTA was able to further significantly separate patients with a high-risk NCCN-IPI score from those with a much better outcome (*n* = 30, 74.3% 3-year PFS rate and *n* = 95, 70.5% 3-year OS rate, respectively) and those with a worse outcome (*n* = 121, 42.6% 3-year PFS rate and *n* = 56, 50.7% 3-year OS rate, respectively; Fig. 4). Notably, although those with an NCCN-IPI score of 4–5 and NCCN-IPI score of 6–8 were significantly different in terms of OS, a high MTA still identified a greater number of patients with OS events than did a high NCCN-IPI (25 and 22, respectively); examples of the tumour burden assessed by the TMTV and MTA based on ^18^F-FDG PET/CT and risk reclassification by the MTA are shown in Fig. 5.

## Discussion

Our data highlight the prognostic significance of MTA, a novel semiquantitative indicator based on ^18^F-FDG PET/CT, in patients with DLBCL in the R-CHOP era. Specifically, (1) MTA and the NCCN-IPI were found to be independent predictors of both PFS and OS before the initiation of R-CHOP therapy in patients with DLBCL, whereas the TMTV was not. (2) Patients with high MTA values have inferior prognoses than those with low MTA values. (3) MTA reclassified patients with a high-risk NCCN-IPI score into a subgroup with a favourable prognosis who should no longer be regarded as high risk. To the best of our knowledge, we are the first to investigate the prognostic significance of MTA and confirm its additive predictive value for prognosis in patients with high-risk NCCN-IPI scores.

The pre-treatment tumour burden is an important predictor of treatment response and disease relapse or progression. However, the commonly used clinical prognosis scoring system, including the NCCN-IPI, only assesses the extent and degree of disease, and the tumour volume is not included. The TMTV represents the tumour burden more precisely than clinical factors such as the Ann Arbor stage and/or LDH level because it measures viable tumour volume [26]. In the past few decades, the prognostic value of the baseline TMTV independent of the IPI for predicting PFS and OS in patients with DLCBL has already been demonstrated in multiple studies [15, 16, 27, 28].

The measurement of the TMTV requires the observer to delineate tumour boundaries with uptake above a chosen threshold; the commonly used thresholds are a fixed SUV of 2.5 [27, 29], a 4.0 [20, 30, 31] or a 41% SUVmax threshold [24, 28, 32, 33]. Ilyas et al. reported that the above methods had similar accuracies in predicting PFS and OS in the same population of 147 patients [34]. Using a 41% SUVmax threshold to compute the TMTV was proven to have the best concordance between the measured and actual volumes, as well as good interobserver consistency [33]; thus, this threshold has been widely adopted. The most accurate segmentation method has not yet reached a consensus. Due to the multitudinous lesions, MTV calculation in lymphoma is time-consuming, restricting its routine clinical implementation [35]. It is still necessary to establish standardised methodologies and accessible indicators suitable for clinical application. Regardless of the total tumour burden, bulky disease, defined as a maximum transverse diameter (MTD) greater than 10 cm of the largest lesion, has always been considered a poor prognostic factor [19]. However, measuring only the MTD of the lesion may underestimate the total tumour burden in patients with diffuse disease. Studies have shown that the prognostic value of the MTD seems to be limited to early lymphoma [36, 37].

We used MTA to assess the prognostic value of DLBCL and found that patients with low MTA had a significantly better prognosis than those with high MTA. In addition, MTA is an independent predictor of PFS and OS, whereas TMTV is not. This may be attributed to necrotic tumour tissue in certain bulky diseases. Necrotic features are defined as visually low uptake surrounded by peripherally high uptake in any tumour lesion on PET/CT. The lesions of DLBCL are usually bulky, multitudinous, and heterogeneous. Larger lesions exhibit greater hypoxia, necrosis, or anatomical and physiological complexity. In the current literature [38–40], tumour necrosis is an important prognostic factor for DLBCL before R-CHOP treatment. Akitoshi Saito et al. [41] reported a greater incidence of tumour necrosis in lymphoma, with extensive necrosis of lymph nodes found in 25% of patients on CT images. The incidence rates of tumour necrosis reported by Hugo J et al. [38, 40] were 13.7% on CT and 20.4% on PET/CT images. Moreover, in the study conducted by Moo-Kon Song et al. [39], the incidence of tumour necrosis on PET/CT images was 18.7%. Although tumour necrosis detected by PET/CT is highly important for prognosis in patients with DLBCL, necrotic lesions do not appear to be included in conventional algorithms at the methodological level of measuring the metabolic burden of tumours via PET/CT. The TMTV, calculated by the 41% SUVmax threshold, corresponds to the part of the tumour with the highest SUV, representing the part of the most metabolically active and aggressive region [42, 43]. However, since ^18^F‑FDG uptake was not observed in necrotic tissue, the necrotic part was not counted in the TMTV; as a result, the true tumour burden was underestimated. From the perspective of PET/CT measurements, MTA appears to represent only the volume or partial metabolic activity of some cancer cells, whereas TMTV seems to represent the overall or total metabolic activity of cancer cells. Therefore, we speculate that the prognostic value of MTA is highly important because MTA calculates the maximum cross-section rather than the entire volume, which reduces the possibility of not being included in the overall tumour burden due to deficiency or decreased metabolism.

On the other hand, massive tumours seem to be more difficult to treat than disseminated tumours, which may be due to the lower permeability of chemotherapeutic drugs to larger tumours, so the metabolic bulk volume (MBV) may have a greater impact on survival than the TMTV. In Delaby et al.’s study [19], patients with high MBV/low TMTV had a 5-year PFS rate of 74%, whereas patients with low MBV/high TMTV had a 5-year PFS rate of 100%. Similarly, Pfreundschuhet et al. [36] reported that patients with massive tumours had worse OS in DLBCL patients treated with immunochemotherapy. Moreover, Tout et al. [44] reported that patients with higher tumour burdens had lower exposure to rituximab, associated with poor treatment response and shorter survival. In summary, the above findings may explain why MTA is more meaningful than TMTV in predicting the prognosis of DLBCL patients in this study.

TMTV was moderately correlated with MTA, explaining why it was not an independent factor in the multivariate analysis in our study. In contrast to our findings, Shagera QA et al. [26] reported that TMTV was an independent prognostic factor for NCCN-IPI. This inconsistency may be due to differences between the populations included in the two studies, with approximately 65% of the enrolled population having advanced-stage disease (stage III/IV) compared with only 58.9% in our study, indicating a lower tumour burden in our study population. Research by Shagera QA et al. also revealed that the TMTV was significantly associated with LDH levels (ρ = 0.67) and was moderately associated with the clinical stage (ρ = 0.45). Our results suggested that MTA was moderately correlated with LDH levels (ρ = 0.514) and weakly correlated with Ann arbor stage (ρ = 0.267). The LDH level is the product of enhanced tumour glycolytic activity and tumour necrosis because of hypoxia, which reflects the tumour growth rate and cellular turnover, and its level depends on the relative proportion of necrotic and viable tissues in the tumour. Large necrotic tumour tend to present elevated LDH levels, but the fraction of viable cells may be low. Therefore, the LDH level could reflect the tumour burden in inpatients with DLBCL [7, 26, 28]. The above findings explain the moderate or strong associations between TMTV, clinical stage and LDH levels in the study of Shagera QA et al. In their study, 60.2% of the patients had elevated LDH levels, whereas only approximately 45.4% of the patients in our study had elevated LDH levels. These findings indicate that the TMTV has a low predictive value in patients with a low tumour burden, whereas the MTA has a greater independent predictive value for this group.

As a widely used metabolic parameter, SUVmax is associated with tumour aggressiveness in previous studies [43, 45]. Gallicchio et al. [46]. reported that, in a small study of 52 DLBCL patients (26 with limited-stage disease and 26 with advanced-stage disease), the SUVmax was the only imaging parameter responsible for predicting PFS compared with the TLG and MTV. There was a longer PFS as the value of the SUVmax increased, with an HR of 0.13 (0.04–0.46). However, their study population was too small to draw this conclusion. In a larger population studied by Ceriani et al., the SUVmax was not an important predictor of survival, which is similar to our findings [47]. One of the reasons for this difference may be that the SUV is a semiquantitative indicator of the FDG metabolic rate and fails to reflect the tumour burden. Furthermore, the time interval between imaging agent injection and image acquisition, partial volume effect, and extravasation of the imaging agent at the injection site may affect the reliability of the SUV.

In clinical practice, we believe that the priority is to accurately identify patients with high-risk diseases because patients with low-risk diseases perform well in current treatment, whereas patients with high-risk diseases require intensive therapy or new treatment regimens, where overexposure to chemotoxic therapy can be avoided. This study confirmed that the MTA and the NCCN-IPI can identify high-risk populations with poor prognoses. More encouragingly, MTA can further stratify high-risk patients with NCCN-IPI scores ≥ 4 into two subgroups with significantly different prognoses. Specifically, it allows for identifying a group of patients with favourable prognoses who should no longer be considered at high risk, and a group of patients with poor prognoses; even if there is a response after R-CHOP, clinicians might consider alternative treatment approaches. As a result, adding MTA to the NCCN-IPI had significant incremental value in predicting the prognosis of DLBCL, especially in high-risk patients with an NCCN-IPI score ≥ 4. Moreover, the current study highlights the benefits of combining NCCN-IPI with MTA in evaluating patients with DLBCL at primary diagnosis.

Since the clinical diagnostic and therapeutic activities of PET/CT are mostly observed through cross-sectional maps, the use of MTA to evaluate the prognostic value of DLBCL is feasible and is relatively simple and rapid to achieve compared with the use of TMTV. It may thus be used as an alternative marker of the TMTV in clinical practice.

This study has several limitations. First, this was a retrospective monocentric study, and the value of this novel and promising parameter needs to be further confirmed in prospective studies with large samples and multiple centres. Second, because ^18^F-FDG PET/CT is not mandatory at baseline evaluation, it is inevitable that patients will have some selection bias in terms of differences in financial status and physician preference. Third, given the convenience and feasibility of the measurement method, only the cross-section for the largest lesion was measured in this study. Perhaps in future studies, we can detect a better prognostic value by using the combined measurements of the maximum cross-sections of all lesions. Finally, no relevant studies have elucidated the exact relationship between the impact of tumour necrosis volume and the TMTV on tumour prognosis, and the relevance of this relationship may need to be further explored in future studies.

## Conclusions

In conclusion, our data suggest that MTA and NCCN-IPI scores are independent predictors of PFS and OS before initiating R-CHOP therapy in patients with DLBCL. MTA has additional predictive value for the prognosis of patients with DLBCL, especially high-risk patients with an NCCN-IPI score ≥ 4. Therefore, pre-treatment with MTA combined with the NCCN-IPI can identify a subgroup with poor survival outcomes and may help guide individualised treatment options.

### Electronic supplementary material

Below is the link to the electronic supplementary material.

Supplementary Material 1

Supplementary Material 2

## Data Availability

The datasets used and/or analysed during the current study are available from the corresponding author upon reasonable request.

## Abbreviations

DLBCL: diffuse large B-cell lymphoma
NHL: non-Hodgkin lymphoma
R-CHOP: rituximab, cyclophosphamide, doxorubicin, vincristine, and prednisone
CR: complete response
NCCN-IPI: National Comprehensive Cancer Network-International prognostic index
^18^F-FDG PET/CT: fluorodeoxyglucose positron emission computed tomography
TMTV: total metabolic tumour volume
TLG: total lesion glycolysis
MTA: metabolic tumour area
PMBCL: primary mediastinal B-cell lymphoma
CNS: central nervous system
LDH: lactate dehydrogenase
ECOG PS: Eastern Cooperative Oncology Group performance status
EN: extranodal involvement
BM: bone marrow
ROI: regions of interest
PFS: progression-free survival
OS: overall survival
ROC: receiver operating characteristic
VIF: variance inflation factor
SD: standard deviation
HR: hazard ratio
CI: confidence interval
