# Supplementary figures and images for: Metabolic tumour area: a novel prognostic indicator based on 18F-FDG PET/CT in patients with diffuse large B-cell lymphoma in the R-CHOP era
Source: BMC Cancer. 2024 Jul 25;24:895. doi: 10.1186/s12885-024-12668-x (PMC11270790; doi:10.1186/s12885-024-12668-x)

**Supplementary Fig. 1:** ROC analysis of MTA for PFS (A) and OS (B).


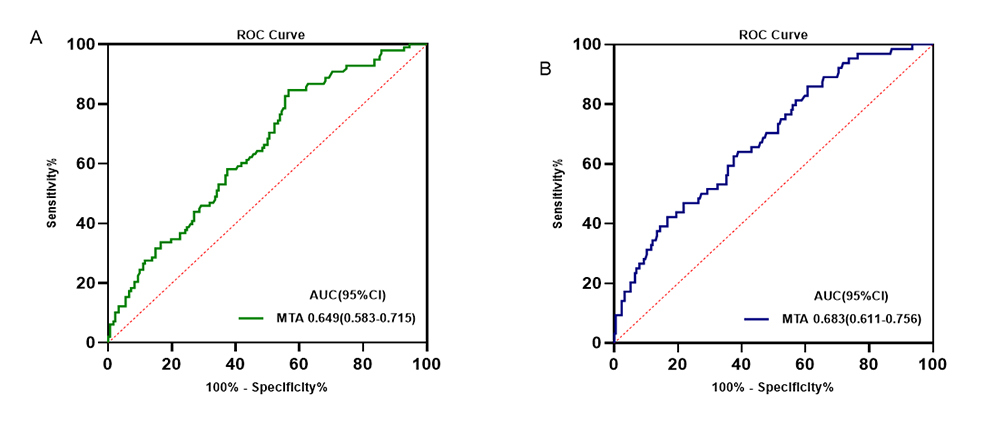

Supplement: Supplementary file 1 — Supplementary Material 1 [file 12885_2024_12668_MOESM1_ESM.docx]
