# Supplementary material for: Metabolic tumour area: a novel prognostic indicator based on 18F-FDG PET/CT in patients with diffuse large B-cell lymphoma in the R-CHOP era
Source: BMC Cancer. 2024 Jul 25;24:895. doi: 10.1186/s12885-024-12668-x (PMC11270790; doi:10.1186/s12885-024-12668-x)
Supplement: Supplementary file 2 — Supplementary Material 2 [file 12885_2024_12668_MOESM2_ESM.docx]

**Supplementary Table 1** Correlation analysis between MTA and other metabolic parameters, NCCN-IPI and components of NCCN-IPI

| Factor | MTA | |
| --- | --- | --- |
|  | Pearson’s correlation coefficient (ρ) | P value |
| TMTV | 0.557 | <0.0001 |
| SUVmean | 0.061 | 0.310 |
| SUVmax | 0.031 | 0.605 |
| Age | 0.134 | 0.025 |
| EN | 0.279 | <0.0001 |
| Ann Arbor Stage | 0.267 | <0.0001 |
| ECOG PS | 0.279 | <0.0001 |
| LDH | 0.514 | <0.0001 |
| NCCN-IPI | 0.442 | <0.0001 |

The Pearson correlation coefficient (ρ) was classified as follows: very weak (ρ=0.0–0.19), weak (ρ=0.20–0.39), moderate (ρ=0.40–0.59), strong (ρ=0.60–0.79), and very strong (ρ=0.80–1.00).
